# Correction: A smile is all you need: predicting limiting activity coefficients from SMILES with natural language processing

**DOI:** 10.1039/d2dd90027k

**Published:** 2022-12-09

**Authors:** Benedikt Winter, Clemens Winter, Johannes Schilling, André Bardow

**Affiliations:** a Energy and Process System Engineering, ETH Zürich Tannenstrasse 3 8092 Zürich Switzerland abardow@ethz.ch; b OpenAI 3180 18TH St San Francisco CA 94110 USA

## Abstract

Correction for ‘A smile is all you need: predicting limiting activity coefficients from SMILES with natural language processing’ by Benedikt Winter *et al.*, *Digital Discovery*, 2022, https://doi.org/10.1039/d2dd00058j.

In the original version of the article, the web link given in the Data Availability Statement for the data repository containing the authors’ datasets and trained models was incorrect. The correct web link to the datasets and trained models is https://polybox.ethz.ch/index.php/s/kyVOt3pwHW26PP4.

The Royal Society of Chemistry apologises for these errors and any consequent inconvenience to authors and readers.

## Supplementary Material

